# The Influence of Cellulose Nanocrystal Characteristics on Regenerative Silk Composite Fiber Properties

**DOI:** 10.3390/ma16062323

**Published:** 2023-03-14

**Authors:** Hak Jeon Kim, Won Jun Lee

**Affiliations:** Department of Fiber System Engineering, Dankook University, Yongin 16890, Republic of Korea

**Keywords:** cellulose nanocrystals, regenerated silk, aspect ratio, crystallinity, composite fibers

## Abstract

Cellulose nanocrystals (CNCs), obtained from natural resources, possess great potential as a bioderived reinforcement for natural-fiber-reinforced composites (NFRPs) due to their superior crystallinity and high aspect ratio. To elucidate the specific parameters of CNCs that significantly affect their mechanical performance, various CNCs were investigated to fabricate high-performance nanocomposite fibers together with regenerated silk fibroin (RSF). We confirmed that the high aspect ratio (~9) of the CNCs was the critical factor to increase the tensile strength and stiffness rather than the crystallinity. At a 1 vol% of CNCs, the strength and stiffness reached ~300 MPa and 10.5 GPa, respectively, which was attributed not only to a stable dispersion but also to alignment. This approach has the potential to evaluate the parameters of natural reinforcement and may also be useful in constructing high-performance NFRPs.

## 1. Introduction

Cellulose nanocrystals (CNCs), needle-like high-crystalline nanoparticles, have been of longstanding interest for renewable composites because of advantages such as their high axial elastic modulus (110~220 GPa), high aspect ratio (3–5 nm wide and 50–500 nm in length), low coefficient of thermal expansion (~25 ppm/K), and low density (1.6 g/cm^3^) [1,2]. The production of CNCs can be achieved using top-down isolation by acid hydrolysis of wood fiber, micro-crystalline cellulose (MCC), micro-fibrillated cellulose (MFC), and nano-fibrillated cellulose (NFC), which leads to highly crystalline (54~88%) cellulose with a high fraction of 1β structure (68~94%) [3]. Recent advances in the selective removal of amorphous segments in cellulose have yielded a scalable production of CNCs [4], which are currently being applied as reinforcement for thermoplastics [5], viscosity modifiers [6], and binders [7].

While the extraction process offers the capacity for mass production, strategies that utilize a wide range of celluloses result in broad size distributions and polymorphs of the CNCs and necessitate a regulatory approach in synthesis in order to utilize the CNCs for composites in a prospective manner [8,9,10]. In particular, the size of the CNCs has an obvious effect on their mechanical properties. For example, wood-based CNCs (4.2 ± 1.2 nm wide and 310 ± 45 nm in length) have a higher aspect ratio than cotton-based CNCs (5.9 ± 1.0 nm wide and 166 ± 34 nm in length), which leads to a high transverse elastic modulus (24.8 ± 7.0 GPa, measured by atomic force microscopy) [11]. Indeed, although CNCs generally retain a cellulose I allomorph after hydrolysis of disordered regions, a cellulose II allomorph can be produced from cellulose I under a restricted reaction, which affects the mechanical properties of the CNCs [10]. Through X-ray diffraction, the elastic moduli of cellulose I and II were 138 and 88 GPa, respectively [12]. However, there is only sparse information on the dependence of mechanical properties of composites on the intrinsic CNCs’ characteristics, such as their diameter, length, purity, and crystalline proportion [13].

Thus far, another challenge for the use of CNCs in composites is how to fully disperse individual crystals in the matrices [14]. Poor interaction between the CNCs and polymeric matrices [15] and a limited set of solvents for suspensions hinder the inter-matrix dispersion of fibrils [16]. Surface modification has emerged as a strong candidate to solve this problem but the modification steps usually require purification with centrifugation and dialysis due to the small size of the CNCs [17]. A good alternative is to use a more biocompatible matrix, for example, regenerated silk fibroin (RSF) [18,19]. Hydroxyl, sulphate, and xanthate groups are abundant in CNCs, thus providing compatibility in aqueous medium and potential hydrogen bonding on the RSF surfaces in composites [20]. Moreover, the RSF shows a substantial mechanical performance together with all types of cellulose including CNCs [21], NFC [22], MFC [23], and MCC [24]. The opportunity to obtain a high mechanical performance composite with the RSF has recently spurred an intensive period of research for various applications such as hydrogels [25], photonic cholesteric films [26], and defect generation [27,28,29]. However, a few studies have considered the effect of CNCs’ characteristics, such as their aspect ratio and loading on composite properties; yet, such studies have been restricted to the CNCs produced by a lab-scale synthesis method and therefore exclude the multitude of variations of CNCs.

This present study presents a comparative analysis of three different CNCs, including LCNC, FCNC, and MCNC, as reinforcements for the RSF composite fibers. Well-dispersed CNCs provided an inherent advantage in the manufacturing of spinning dope for wet spinning, which enables the production of composite fibers. Furthermore, by utilizing a well-established wet-spinning technique with hot-drawing procedures with the CNCs, the alignment and crystallization of the RSF were maximized, which is known to enhance composite axial properties. To determine the key parameters of the CNCs in composite fibers, the diameter, length, morphology, crystal structure, impurity amount, and chemical functional group of all of the CNCs were analyzed and then correlated to their tensile mechanical performance.

## 2. Materials and Methods

### 2.1. Materials

Commercially available CNCs produced from different manufacturers were purchased directly from Celluforce (FCNC, Montreal, QC, Canada), Cellulose Lab (LCNC, Toronto, ON, Canada), and the University of Maine (MCNC, Orono, ME, USA). The silkworm Bombyx mori was purchased from Uljinfarm (Uljin, South Korea). Sodium oleate (22295-1201) was purchased from JUNSEI Chemical Co., Ltd. (Extra pure, Tokyo, Japan). Sodium carbonate anhydrous was purchased from Samchun Chemical Co., Ltd. (>99.0% Extra pure, Gyeonggi-do, Republic of Korea). Trifluoroacetic acid (TFA) was purchased from TCI Chemical Company (>99.0%, Tokyo, Japan). All of the employed chemicals and materials were directly used without further purification.

### 2.2. RSF Preparation: Degumming of the Bombyx Mori Cocoon

The degumming process, to discard the sericin in the silkworm, proceeded as follows: (1) each cocoon was cut into quarters and gently washed in distilled water at 65 °C and then transferred to a desiccator at 10% relative humidity and dried for 3 days at room temperature; (2) the dried cocoons were immersed into an alkaline soap solution consisting of 0.1% (*w*/*v*) of sodium oleate and 0.067% of sodium carbonate aqueous solution and boiled at 100 °C for 1 h; (3) the solution was filtered with a non-woven filter and dried in a desiccator for 3 days at 10% relative humidity at room temperature [30].

### 2.3. Preparation of Spinning Dopes

Spinning dopes were prepared with a constant total solids content (CNC and RSF) of 112 mg/mL in TFA solvent. The RSF was initially dissolved in TFA (25 °C for 30 min). The supplied CNC gel or the suspension from Cellulose Lab, Celluforce, and the University of Maine was freeze-dried initially and collected in white powder form. A specific amount of CNCs was dispersed in TFA with ultrasonication (JAC 3010, 150 W) for 20 min with different concentrations. CNC solution in different concentrations was added to the RSF solution and stirred at RT for 10 min. The mixture was briefly sonicated for 5 min and passed through a syringe filter (PTFE, 200 µm) to discard residual agglomerates.

### 2.4. Fabrication of CNC/RSF Composite Fibers

The CNC/RSF composite fibers were spun through injection into a customized wet-spinning apparatus at room temperature. All of the spinning dopes were extruded into a methanol coagulation bath using a syringe pump (KDS100, KD scientific, Holliston, MA, USA) through a 26-gauge spinneret at 2 mL/h of injection speed without high-voltage supply (i.e., classic coagulation spinning technique). The composite fibers were soaked in a coagulation bath for 6 h, collected, and wound about a circular reel [31]. The fibers were hot-drawn under tension between two drum winders, with the initial winder speed set to 20 cm/min and the final speed set to 40 cm/min, resulting in a draw ratio of 1:2, whilst travelling through a split furnace (50 cm in length) operated at 120 °C (Figure S6) [32].

### 2.5. Characterization of CNCs 

Atomic force microscopy (AFM) observations were performed on Park Systems XE-100 equipment to evaluate the morphology of the CNCs, including their width, length, and aspect ratio. A drop of diluted CNC aqueous suspension (c.a. 0.01 vol%) was spin-coated on the SiO_x_ substrate after an O_2_ plasma treatment for 5 min and then air-dried. AFM images were obtained under ambient conditions in the tapping mode. High-resolution transmission electron microscopy (HR-TEM) observations were performed on a JEOL JEM-2100F operating at 200 kV. To observe the CNCs clearly, a drop of dilute (c.a. 0.01 vol%) CNCs was deposited on a copper grid coated with a carbon support film. The specimen was then negatively stained with a drop of UranylLess lead citrate. Fourier transform infrared spectroscopy (FT-IR, Perkin Elmer, Waltham, MA, USA, Spectrum Two) was used to characterize the surface functional groups of the CNCs using an attenuated total reflection (ATR) accessory with a non-destructive measurement method. All of the samples were scanned within the wave range of 650~4000 cm^−1^. Thermogravimetric analysis (TGA, Scinco, Seoul, Republic of Korea, N1000) was performed in a N_2_ atmosphere (flow rate of 30 mL/min), isothermally held for 30 min at 100 °C to discard residual water before heating from 100 °C to 500 °C at 10 °C/min. Differential scanning calorimetry (DSC, Perkin Elmer, DSC 4000) was used to characterize the thermal properties of the CNCs. Dry N_2_ was used as a purge gas at a rate of 20 mL/min during the thermal treatments. A 3 mg amount of CNCs was placed in an aluminum pan. The heating cycle’s temperature was increased from 25 °C to 400 °C at 10°C/min. The crystallinity of the CNCs was determined by a Rigaku SmartLab MXD10 with CuKα radiation (λ = 1.54056 Å) generated at 40 kV and 30 mA. A 1 mg amount of CNCs was mounted onto a quartz substrate. Scans were obtained in the range of 2θ = 10~50° at a scan rate of 0.1°/s and analyzed using ICDD reference data. The crystallinity index (CI) was calculated from the height ratio between the intensity of the crystalline peak (I_200_-I_amorphous_) and the total intensity (I_200_) after the subtraction of the background signal measured without the CNCs.

### 2.6. Characterization of CNC/RSF Composite Fibers 

The morphology of the composite fibers was characterized using scanning electron microscopy (SEM, Hitachi, Tokyo, Japan, S-5200) with the conditions of 10 kV and 10 µA with Pt sputtering. Small-angle X-ray scattering (SAXS) analysis was implemented to characterize the overall alignment of the composite fibers at the Pohang Accelerator Laboratory (PAL) on a 4C beamline with an X-ray beam wavelength of 0.675 Å at a sample–detector distance of 1 m. The individual CNC alignment in the composite fibers was obtained from bundles of parallel fibers using wide-angle X-ray scattering (WAXS, Bruker, Billerica, MA, USA, D8 Discover). The fiber specimens were mounted perpendicular to the X-ray beam. The scattering patterns of the fibers were recorded with a connected image plate system. The alignment was quantified by a dedicated azimuthal scan. A step width of 0.2° at 5°/min was used for the azimuthal scan from 0 to 180°, with fixed 2θ = 22° corresponding to the (200) plane of the cellulose crystal. The full width at half-maximum (FWHM) of the azimuthal intensity distribution at 2θ = 22° was characterized by Lorentzian distribution. The tensile properties of the single composite fibers were determined following the British standard (BS ISO 11566:1996). The fibers were loaded onto cardboard holders, with gauge lengths of 15 mm ± 0.5 mm, using an epoxy adhesive (Araldite Rapid 24 mL). The samples were tested using an Instron 3365 model fitted with a 10 N load cell. The samples were measured with a cross-head displacement rate of 1 mm/min until failure. The fiber cross-sectional areas were obtained from the SEM fracture surfaces to calculate the true stress.

## 3. Results and Discussion

### 3.1. Properties of CNCs That Determine Their Mechanical Performance

**Figure 1:**
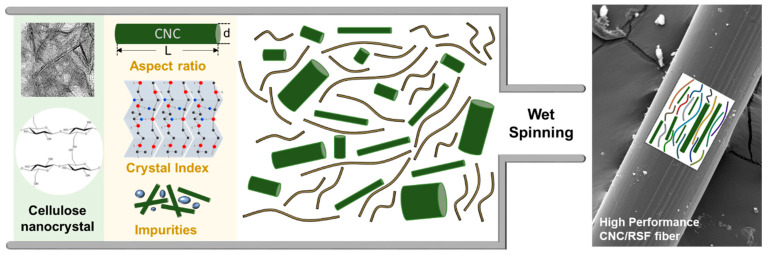
Illustration of fiber formation with CNC and RSF. Three different characteristics of CNC, such as (1) aspect ratio, (2) crystal index, and (3) impurities, influence the properties of the composite fiber including alignment and crystallinity, resulting in different mechanical features.

To identify the fundamental parameters of the CNCs that determine their mechanical performance, we carried out an extensive evaluation of the CNCs including: (1) atomic force microscopy (AFM); (2) transmission electron microscopy (TEM); (3) thermogravimetric analysis (TGA); (4) Fourier transform infrared spectroscopy (FT-IR); (5) differential scanning calorimetry (DSC); and (6) X-ray diffraction (XRD), produced by three different worldwide suppliers (Table S1). Note that the CNC products on the lab bench, on the small scale, were excluded for the sake of clarity. The properties of the CNCs are governed by several aspects including: (1) their aspect ratio (i.e., the ratio between their length and their width); (2) the crystal index; (3) the surface functionality; and (4) the number of impurities. The effect of varying the properties of the CNCs, as a filler, was examined for the mechanical composite fiber produced with regenerated silk fibroin (RSF) as a matrix (Figure 1). Considering the resistance to mechanical deformation and the complementary design for reinforced bio-composites, the association between the CNCs and RSF can guide the appropriate use of CNCs with different properties. In particular, the predominant hydrophobic (Gly-Ser-Gly-Ala-Gly-Ala)_n_ sequences of RSF, prone to folding into β-sheet structures through inter- and intra-molecular hydrophobic interactions and hydrogen bonding, have shown excellent compatibility and mechanical properties in the previous literature [28,29].

### 3.2. Estimation of Aspect Ratio of CNC and Their Agglomeration

**Figure 2:**
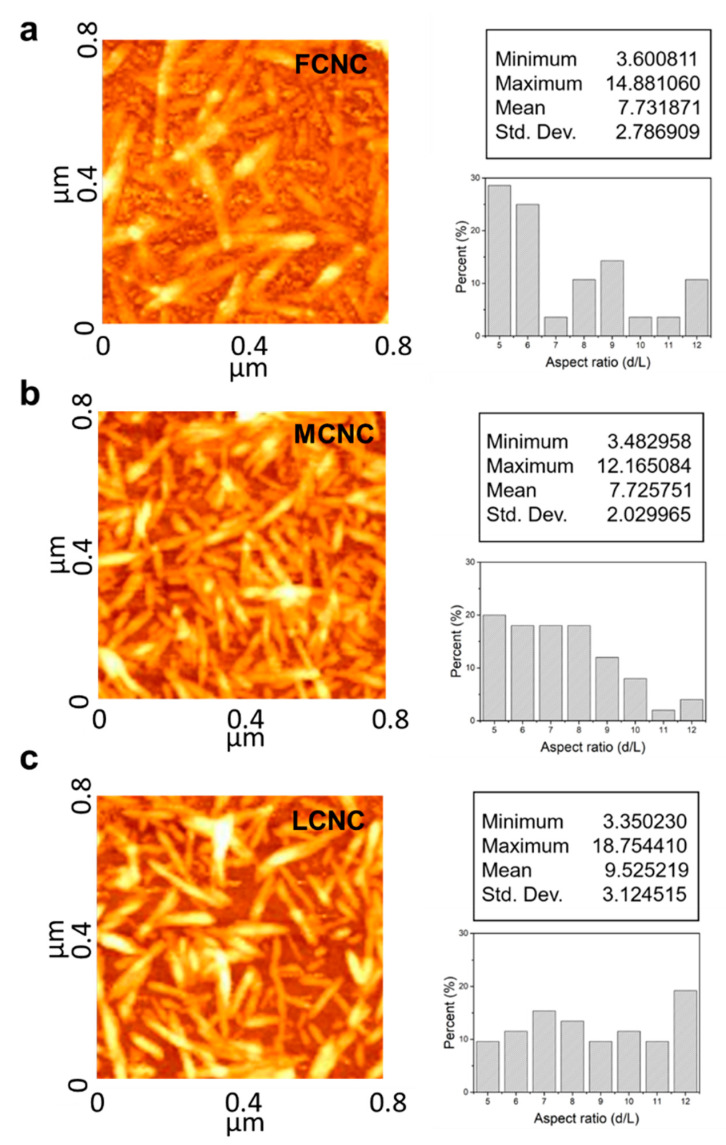
AFM topographic images (on the left) and histograms of the aspect ratio (on the right) from various CNC sources. AFM image shows that the majority of individual CNCs are well dispersed, which provide a direct comparison in nanoscale. (**a**) FCNC, (**b**) MCNC, and (**c**) LCNC.

**Figure 3:**
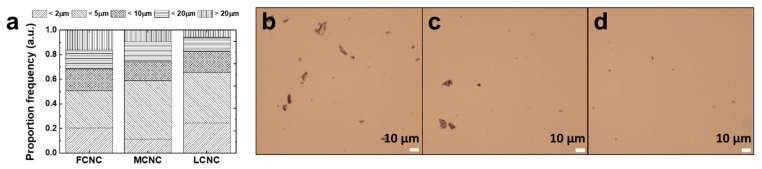
CNC cluster frequency proportions from a variety of CNC samples. (**a**) The bar graphs depict the respective frequency proportions for FCNC, MCNC, and LCNC. Optical microscopic images of (**b**) FCNC, (**c**) MCNC, and (**d**) LCNC suspension with a concentration of 1 wt%.

CNCs contribute to the composite mechanical stiffness with an effective modulus that decreases with their aspect ratio [30]. To evaluate the aspect ratio of various CNCs, we used two-step methodologies. First, the freeze-drying step was carried out to discard water from the as-received samples. Note that commercial CNCs are typically available in the form of suspensions with a variety of concentrations. Then, an appropriate amount of DI water was introduced to produce a diluted suspension (c.a. 0.005 wt%) with the aid of magnetic stirring. It should be noted that all of the CNC samples here were diluted to provide a direct comparison of their aspect ratios with a low degree of agglomeration. Three CNC samples—namely FCNC, MCNC, and LCNC—were then subjected to AFM investigation. Around 10 µL of CNC suspension was spin-coated on a silicon wafer (1 cm^2^) at a speed of 3500 rpm. The AFM section views are shown in Figure 2 (left). Indeed, the quantitative analysis of the AFM images determined their aspect ratio from the length and diameter distributions (Figure S1). The average aspect ratio of the FCNC was 7.73 with an overall standard deviation of 2.78. One particular feature of the FCNC is that additional salt was observed between the CNC rods. Note that additional salt may aid in the separation of CNCs [31]. When the diameter of the CNCs is more than 30 nm or even larger, the formation of a cluster may occur. Indeed, on the basis of the optical microscopy images (Figure 3), we also confirmed that there was a larger fraction of agglomerates over 20 µm (>19%) of FCNC compared with the MCNC and LCNC for the 1 wt% CNC suspension. The average aspect ratios of the MCNC and LCNC are 7.72 and 9.52 with overall standard deviations of 2.02 and 3.12, respectively. The higher aspect ratio of the LCNC may be related to (1) the degree of acid hydrolysis, (2) the cellulose source, and (3) the ionic strength. Note that previous studies have identified various aspect ratios of CNCs with different approaches [32,33].

With regard to the direct reinforcing ability of CNCs with high aspect ratios, previous studies have explored the negative effect of agglomeration [12]. However, critical factors that determine the degree of dispersion are still poorly understood. In principle, the reasons for creating local CNC agglomerations are their high aspect ratio and surface energy and their low bending rigidity. To quantitatively evaluate the impact of their aspect ratio, a diluted aqueous CNC droplet (c.a. 0.1 wt%) was investigated (Figure 3). In brief, droplets were gradually deposited on the center of 500 rpm rotating mica substrates. Images were taken at a constant magnification (20X) and analyzed using the software Image J (version 1.53t). The size of the agglomerates was estimated using the agglomerate area ratio (the agglomerates were defined as domains with a selected circle diameter of 1. <2 µm, 2. <5 µm, 3. <10 µm, 4. <20 µm, and 5. >20 µm). The agglomerate area ratios under 5 µm were quantified to be 0.50, 0.58, and 0.61 for the FCNC, MCNC, and LCNC, respectively. The results showed no significant difference in the agglomerate area ratio under 5 µm, thus indicating that the aspect ratio, at least when they are diluted, does not play a critical role in agglomeration. Interestingly, the FCNC showed a 0.18 agglomerate area ratio over 20 µm in contrast to the MCNC (0.09) and the LCNC (0.04). This result may be related to the presence of additional salt, as observed above in AFM (Figure 2). The ionic strength of CNCs could be increased by additional sulfates of the respective cation, triggering agglomeration.

### 3.3. Crystalline Structure of CNC with Various Allomorphs

The microstructure of the CNCs was characterized with wide-angle X-ray scattering (WAXS) to identify their crystalline index (CI) and polymorphs (cellulose I and cellulose II). Inherently, CNCs have a cellulose Iβ structure, which is monoclinic with parameters a = 7.78 Å, b = 8.20 Å, c = 10.38 Å, and β = 96.5°. Indeed, another allomorph of monoclinic cellulose II (a = 8.10 Å, b = 9.03 Å, c = 10.31 Å, and β = 117.1°) can be obtained by NaOH swelling or by chemical regeneration. Note that the allomorph of cellulose III is excluded in our estimation due to the lack of ammonia and various amines in the process.

**Figure 4:**
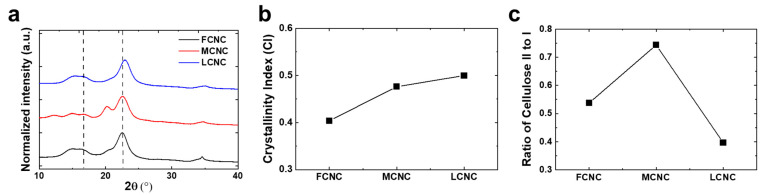
(**a**) 1D wide-angle X-ray scattering (WAXS) profiles of various CNCs, (**b**) crystallinity index, and (**c**) the ratio of cellulose II to cellulose I of CNCs. To calculate the crystallinity index and the ratio, the peak separation was used to separate the background and overlapping peaks of the 1D WAXS integral curves. Dotted lines represent (left) amorphous, and (right) (200) plane of cellulose I, respectively.

Figure 4a shows the WAXS profile of bulk powder for a variety of CNCs. All of the CNCs exhibited characteristic scattering peaks at 2θ = 16.5° and 22.6°, corresponding to the (110) and (200) planes, which were indexed with a cellulose I structure. Notably, the WAXS profile obtained from the MCNC showed a characteristic peak at 2θ = 20.0°, corresponding to the (110) plane of cellulose II, which helps to identify the ratio of cellulose II to cellulose I below. The relative crystallinity index (*CI*) (i.e., the ratio of the sum of the crystalline peak areas to the total area) was calculated in order to obtain numerical numbers using the Segal method using the following equation.

(1) $$CI=\frac{I_{200}-I_{amorphous}}{I_{200}}$$

where *I*_200_ and *I_amorphous_* are the intensity at 2θ = 22.6° and the lowest intensity between the (200) and (110) peaks around 2θ = 18°, respectively. The CIs of the CNCs were 0.67, 0.64, and 0.66 for the FCNC, MCNC, and LCNC, respectively. Note that the *CI* is directly related to the degree of acid hydrolysis and production conditions controlled by the supplier, which is not the focus of this study. A slight increase in the *CI* of the FCNC was observed, which may be due to the aid of additional salt. The supporting thermal analysis with DSC and TGA indicated that the FCNC exhibited excellent thermal stability (i.e., its melt temperature and decomposition temperature) (Figure S2). Because the crystallite size decreases from bulk cellulose to nanocrystals, the main peak of cellulose I (i.e., 2θ = 22.6°) became broader after hydrolysis, enabling us to estimate the crystalline domain size. Note that the crystalline domain size does not correspond to the particle size, as CNCs contain multiple crystalline domains. Analyzing the crystallite size of the (200) plane using the Scherrer equation (see below) shows the linear dependence on the crystalline index [33].

(2) $$Crystallitesize\left(\tau \right)=\frac{K\lambda }{\beta cos\theta }$$

where *K* (0.91), *λ* (1.54060 Å), and *β* are the Scherrer constant, X-ray wavelength, and the width of the peak at half of its height, respectively. The crystallite sizes were calculated to be 5.48, 4.38, and 5.00 for the FCNC, MCNC, and LCNC, respectively (Table 1).

We also hypothesized that the crystal structure of cellulose may play a critical role in determining whether crystalline CNCs would be able to reinforce composites effectively. In principle, cellulose I has eight hydrogen bonds per glucose unit, whereas amorphous cellulose contains 5.3 hydrogen bonds. This results in the amorphous and crystalline cellulose differing in their long-range structural arrangement, which affects their mechanical properties. Note that the stiffness of cellulose I (c.a. ~138 GPa) is higher than cellulose II (c.a. ~88 GPa) [12]. Plotting the ratio of I_200,celI_ (i.e., the (200) plane of cellulose I, 2θ = 22.6°) to I_110,celII_ (i.e., the (110) plane of cellulose II, 2θ = 20.0°) was exploited to estimate the relative amount of cellulose II polymorph (Figure 4c). Note that the (110) plane has the strongest peak of the cellulose II polymorph [10]. The ratios were estimated to be 0.43, 0.67, and 0.40 for the FCNC, MCNC, and LCNC, respectively. The Fourier transform infrared (FT-IR) spectra also showed that the band at 898 cm^−1^ was noticeably higher in the MCNC (Figure S4). Note that the band at 898 cm^−1^ represents the C-O-C in-plane stretching of β-linked glucose residues, which is well observed in the cellulose II polymorph.

### 3.4. Reinforcing Ability of Various CNCs

**Figure 5:**
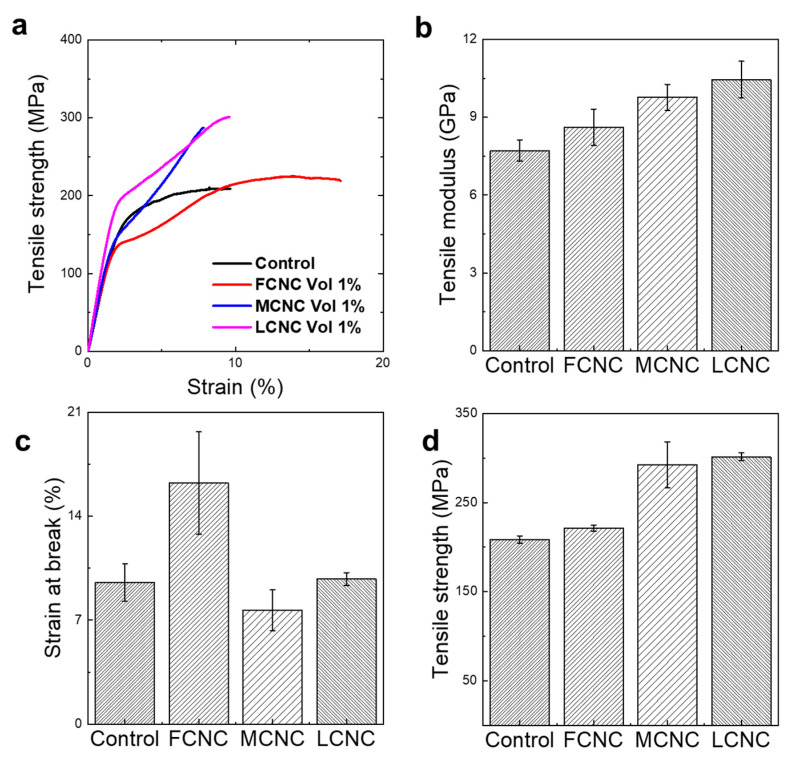
Influence of characteristics on mechanical strength of composites reinforced by three different CNCs. Different characteristics of CNC affects tensile modulus, strain at break, and tensile strength in different level. (**a**) Characteristic stress-strain curves, (**b**) Bar diagram of tensile modulus, (**c**) Bar diagram of strain at break, and (**d**) Bar diagram of tensile strength.

Given that the crystal structure and aspect ratio are important in influencing the mechanical characteristics of CNCs, we hypothesized that CNCs might have different reinforcing abilities in composites (Figure 5). Basically, the mechanical performance of composite fibers is determined by the properties of the reinforcement, the matrix, and their interfacial adhesion [34]. To elucidate the effect of CNCs as a reinforcement, we defined the matrix of the regenerated silk fibroin (RSF) and a 1% volume fraction of reinforcement. Note that RSF was chosen as a matrix because of its natural compatibility and well-known characteristics. To prepare the composite fibers, a 1 vol% of CNC was mixed with RSF in trifluoroacetic acid (TFA) to form a spinning dope, and then spun into a bath filled with methanol coagulants. All of the composite fibers were hot drawn at 120 °C, to a draw ratio of 2, below the CNC decomposition temperature of 180 °C. After visualization with optical and scanning electron microscopy, we confirmed that all of the spinning dopes were well dispersed and fabricated into a continuous fiber with a diameter of ~ 70 µm. Remarkably, the LCNC that reinforced the RSF exhibited significant mechanical characteristics, with a stiffness (E) of 10.45 ± 0.71 GPa and a strength (σ) of 301.37 ± 4.48 MPa at a strain at break of 9.76%. The results were comparable to those of the bare RSF fiber (σ = 208.62 ± 4.03 MPa and E = 7.71 ± 0.41 GPa), the FCNC/RSF composite fiber (σ = 221.34 ± 3.62 MPa and E = 8.62 ± 0.70 GPa), and the MCNC/RSF composite fiber (σ = 292.39 ± 25.84 MPa and E = 9.77 ± 0.50 GPa). A comprehensive table of the tensile modulus, strength, and strain at break from the RSF composites with CNCs is provided in Table 2. We focused on which features of the CNCs affected the stiffness and toughness rather than on how the CNCs reinforced the composites. Remarkably, it is obvious that the high aspect ratio of the LCNC provides an excellent reinforcement ability for the deformation of the composites. Indeed, while the interfacial adhesion is difficult to define in this study, the degree of dispersion is expected to contribute to the mechanical features of composites. Specifically, we observed poor dispersion of the FCNC in the spinning dope, which was critically ineffective for mechanical performance with poor interfacial adhesion. In addition, the failure strain in the FCNC-based composites is clearly higher than the other composites due to the non-uniform distribution of the CNCs. Since cellulose Ⅲ has been previously reported to be amorphous, [35] cellulose Ⅲ was excluded to verify the effect. Regarding the ratio of cellulose II to cellulose I, a high amount of cellulose II in the MCNC compared with the LCNC was shown to be less effective as reinforcement. Although the CI and the amount of cellulose II may be critically important to the stiffness of the reinforcement, we confirmed here that these factors were not the primary causes of the difference in the stiffness of composites.

### 3.5. Reinforcing Ability with Different Volume Fractions and Alignment Effect

**Figure 6:**
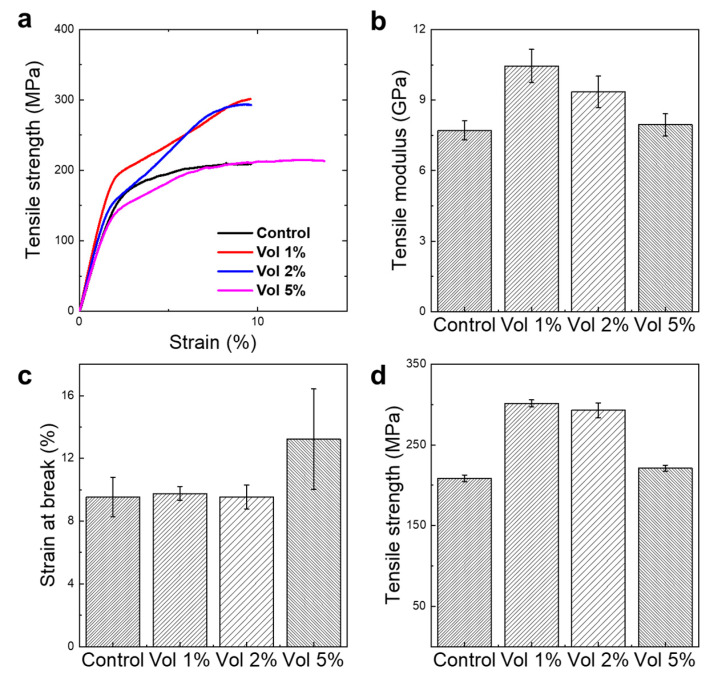
The mechanical properties of regenerated silk fibroin fiber reinforced by LCNC as a function of volume fraction. Different amounts of CNC affects tensile modulus, strain at break, and tensile strength. (**a**) Characteristic stress-strain curves, (**b**) Bar diagram of tensile modulus, (**c**) Bar diagram of strain at break, and (**d**) Bar diagram of tensile strength.

For the comparison of the mechanical properties depending on the alignment effect and volume fraction of CNCs, we conducted tensile mechanical tests, as depicted in Figure 6. The mechanical properties are highest at vol 1% of CNC reinforcement. While there was no significant difference between vol 1% and 2%, the increase rate to the first transition region, called the yield point, is significantly higher than that of vol 2%, which confirms that the stress transfer from the matrix to the reinforcement works effectively at vol 1%. Note that the interfacial stress between the reinforcement and the matrix could be hampered by increasing the volume fraction of the reinforcement due to the agglomeration. Notably, the tensile strength and Young’s modulus with vol 5% of CNCs was similar to or lower than the fiber of the control, which indicated that the LCNC was agglomerated and hinders effective stress transfer between the CNCs and RSF. The strengthening mechanism could also be observed with the alignment effect and the 2D-WAXS was also investigated for the purpose.

**Figure 7:**
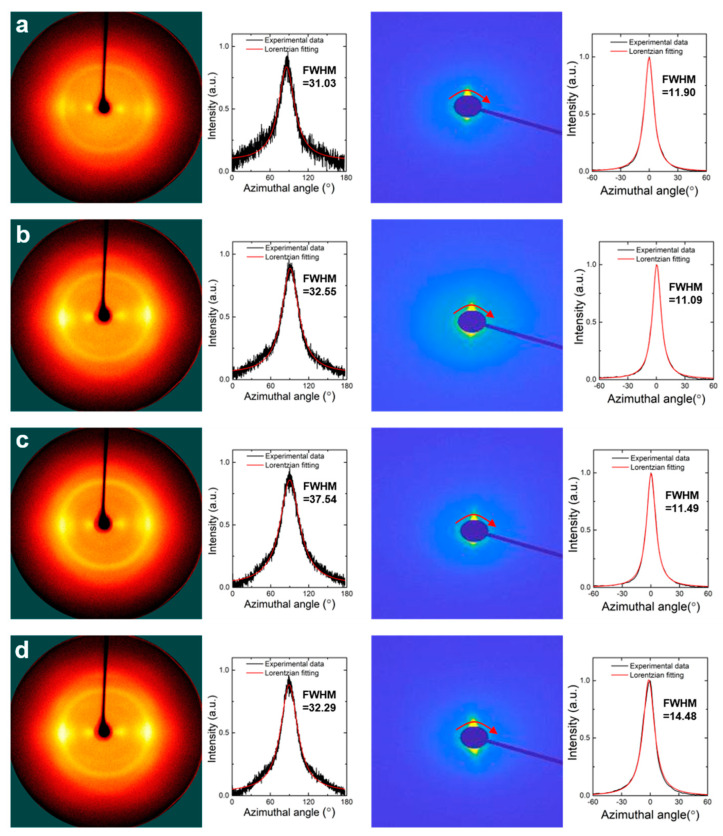
WAXS 2D pattern and angular scattered spectra (left) and SAXS 2D pattern and angular scattered spectra (right); (**a**) RSF for control, (**b**) RSF with vol 1% of LCNC, (**c**) RSF with vol 2% of LCNC, and (**d**) RSF with vol 5% of LCNC.

To identify the alignment effect of the CNCs along the fiber axis in the nanocomposite fiber, 2D patterns and their azimuthal scattering spectra were obtained from WAXS and SAXS, respectively (Figure 7). WAXS and SAXS were utilized to observe the orientation of the RSF matrix and CNC reinforcement, respectively. Note that all of the samples were measured with the LCNC-reinforced RSF with different volume fractions after hot-drawing (i.e., a draw ratio = 2). Nanocomposite fibers with the FCNC and MCNC were excluded so as not to include other features of the CNCs such as crystallinity and impurities for the sake of clarity. Interestingly, the full-width half maximum (FWHM) values from WAXS with different volume fractions confirm that the stretching of the RSF chains was less affected by the addition of the CNCs. However, the FWHM values from SAXS suggest that an adequate addition of CNCs is necessary to maintain or increase the alignment of the CNCs along the fiber axis.

(3) $$f=\frac{3<{cos}^{2}\delta >-1}{2}$$

The Herman’s orientation factor values calculated by Equation (3) were 0.7216, 0.7222, 0.7213, and 0.7134 as a function of the volume fraction, respectively (control, vol 1%, 2%, and 5%). The increase between vol 1% and vol 2% of CNCs was mainly attributed to the aggregation of the CNCs, which was confirmed by a cross-sectional image of the nanocomposite fibers. As noted in Figure S4, when a 5 vol% of CNCs was introduced, a bold line due to the agglomeration of the CNCs was clearly observable, which was confirmed from the dope solution as well. The high concentration of CNCs could induce internal percolation and deteriorate the mechanical performance with nano-clustering. This implies that the regularity and uniformity of CNCs may be critical to fabricate future natural-fiber-reinforced composites (NFRPs) as well as a high aspect ratio.

## 4. Conclusions

In summary, we evaluated the mechanical properties of a nanocomposite fiber composed of RSF as the matrix and CNCs as the reinforcement. To assess the critical parameter of the CNCs in composites, their morphology, impurity level, crystallography, and chemical features were analyzed with AFM, TEM, XRD, DSC, and FT-IR spectroscopy. Most importantly, the high aspect ratio of CNCs appears to be very prominent in order to obtain a high mechanical performance with excellent alignment. Note that their dispersion should be warranted at a certain level from an early experimental stage in spinning dope preparation. The crystallinity and the crystal structure (cellulose I and cellulose II) have less impact on mechanical performance after mechanical stretching with heat on a macroscopic scale. Additionally, the addition of salt for the CNCs’ dispersion issue might be helpful for stable dispersion in a water medium; however, it potentially causes interfacial issues between the CNCs and the matrix, which was observed in their mechanical performance and microscopic images. The improvement in tensile strength (~300 MPa) and stiffness (~10.5 GPa) suggests that CNCs could be a strong candidate for effective reinforcement in NFRPs. The overall performance obtained here demonstrates that further observation should be pursued to analyze their micro-mechanical behavior.

## Figures and Tables

**Table 1:** Crystallite size & Crystallinity index of various CNCs.

	FCNC	LCNC	MCNC
Crystallite size, τ (nm)	5.48	5.00	4.38
Crystallinity index	0.61	0.66	0.61

**Table 2:** Mechanical properties of different CNC types in nanocomposite fiber.

	Bare RSF	FCNC/RSF	MCNC/RSF	LCNC/RSF
Modulus (GPa)	7.71 ± 0.41	8.62 ± 0.70	9.77 ± 0.50	10.45 ± 0.71
Strength (MPa)	208.62 ± 4.03	221.34 ± 3.62	292.39 ± 25.84	301.37 ± 4.48
Strain at break (%)	9.53 ± 1.26	16.24 ± 3.45	7.68 ± 1.37	9.76 ± 0.44

## Data Availability

The data presented in this study are available on reasonable requisition.

## Supplementary Materials

The following supporting information can be downloaded at: https://www.mdpi.com/article/10.3390/ma16062323/s1, Table S1: Representative various CNCs used in this study; Figure S1: Diameter and length histograms of various CNCs; Figure S2: Thermal stability and thermograms of various CNCs; Figure S3: The dope solution and cross-sectional images of composite fiber with different volume fractions of CNCs: (a) control, (b) vol 1%, (c) vol 2%, and (d) vol 5%; Figure S4: FT-IR spectra of various CNCs; Figure S5: TEM images of various CNCs.
