# Supplementary material for: The Influence of Cellulose Nanocrystal Characteristics on Regenerative Silk Composite Fiber Properties
Source: Materials (Basel). 2023 Mar 14;16(6):2323. doi: 10.3390/ma16062323 (PMC10052345; doi:10.3390/ma16062323)
Supplement: Supplementary file 1 [file materials-16-02323-s001.zip › materials-2250736-supplementary.pdf]

Supplementary Materials

Influence of Cellulose Nanocrystal Characteristics on Regenerative Silk Composite Fiber Properties

Hak Jeon Kim [1], and Won Jun Lee [1,*]

1 Department of Fiber System Engineering, Dankook University, Yongin, 16890, Republic of Korea
* Correspondence: wjlee@dankook.ac.kr Tel.: +82-31-8005-3563

Table S1. Representative various CNCs used in this study.

	FCNC	MCNC	LCNC
Country	Canada	U.S.A.	Canada
Suppliers	CelluForce	Maine Univ	CelluloseLab

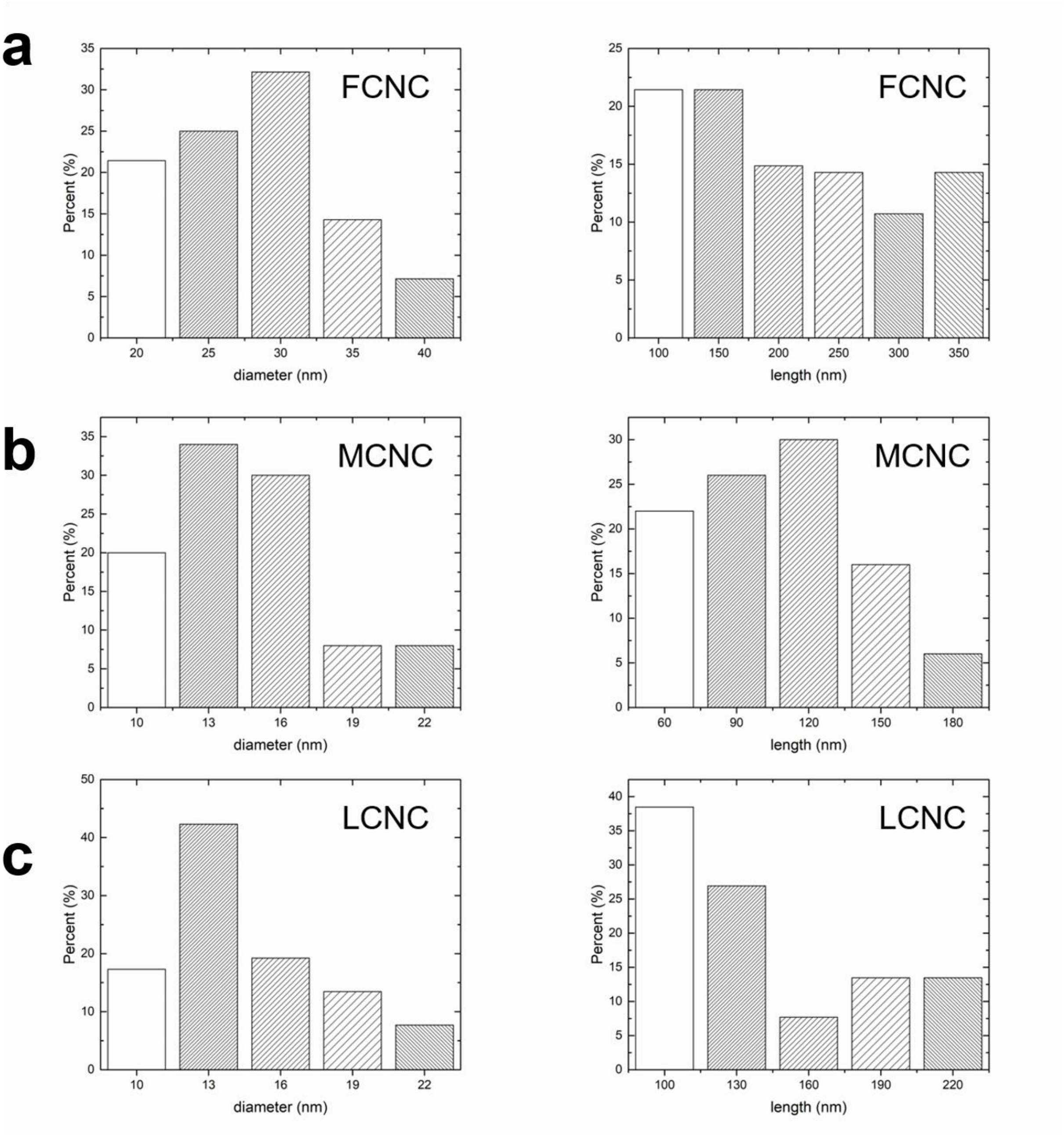

Figure S1. Diameter and length histograms of various CNCs

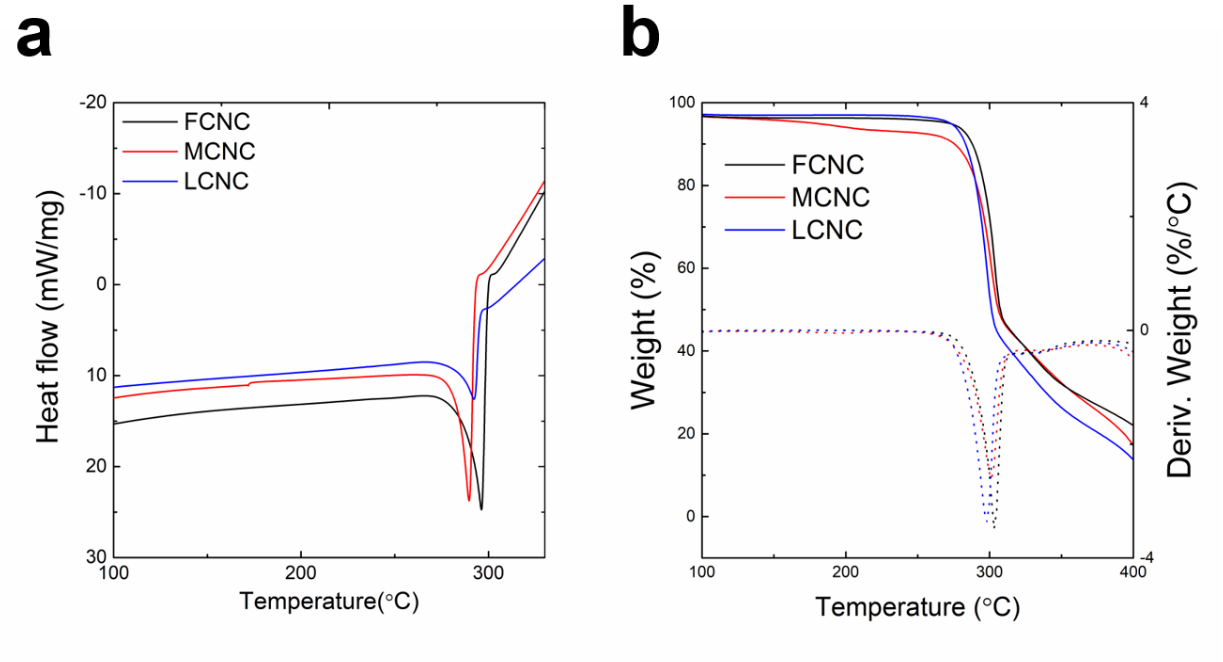

Figure S2. Thermal stability and thermograms of various CNCs.

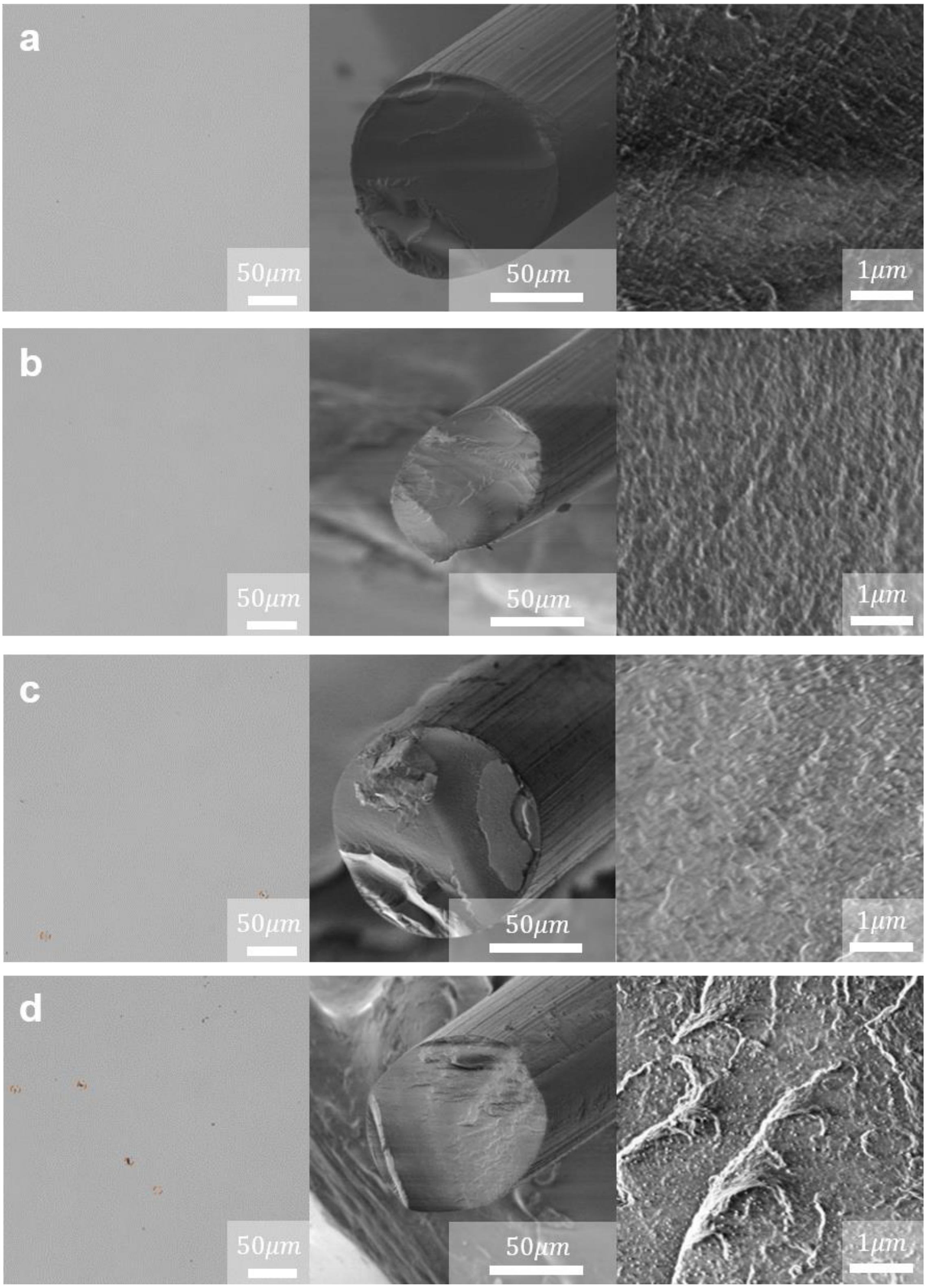

Figure S3. The dope solution and cross-sectional images of composite fiber with different volume fractions of CNCs : a) control, b) vol 1%, c) vol 2%, and d) vol 5%.

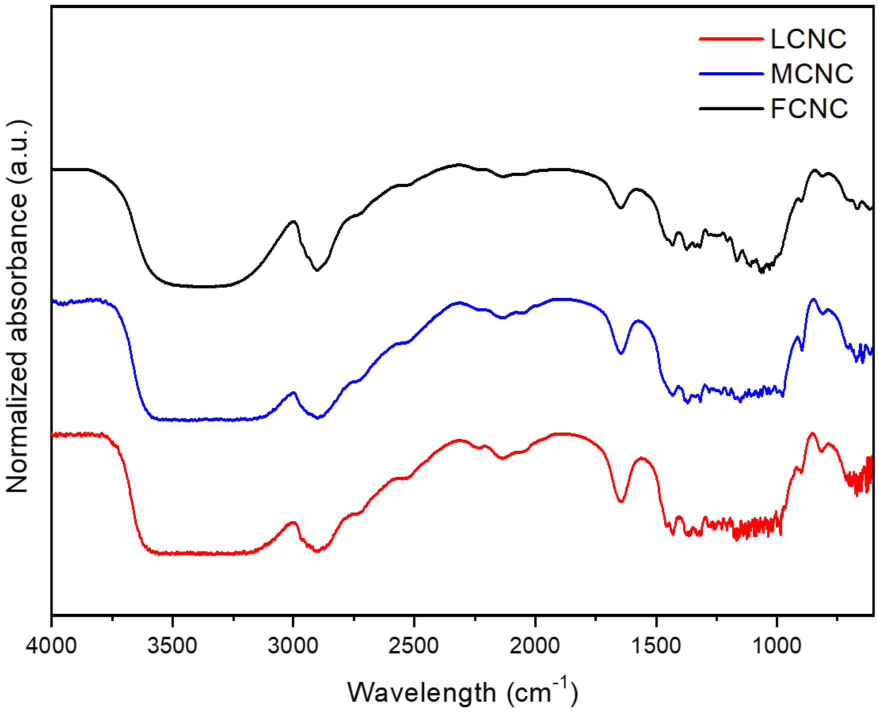

Figure S4. FT-IR spectra of various CNCs.

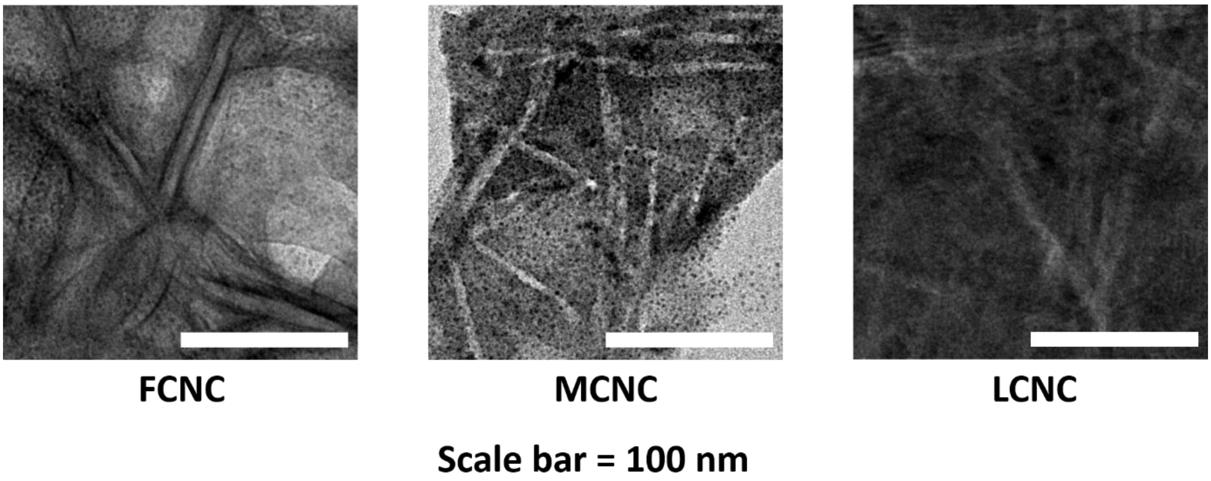

Figure S5. TEM images of various CNCs

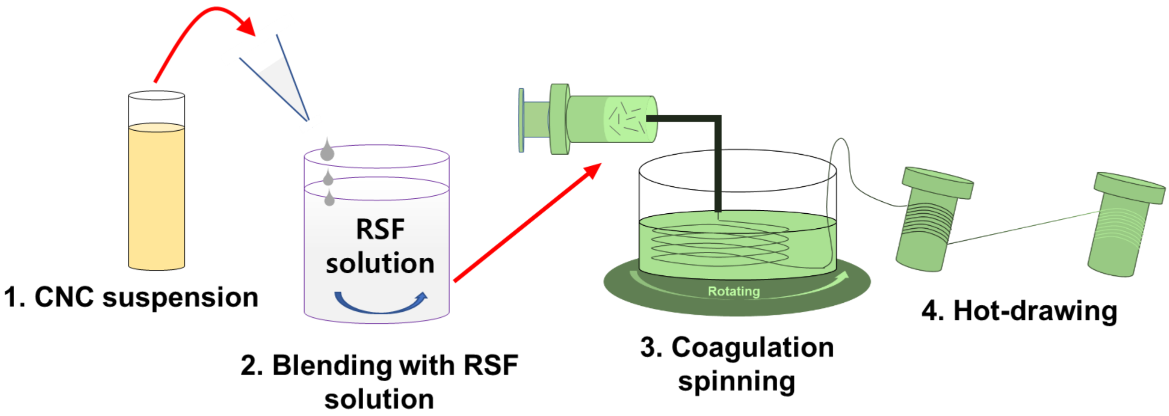

Figure S6. Schematic illustration of RSF/CNC composite fiber spinning
